# Crystal structures of two C,N-disubstituted acetamides: 2-(4-chloro­phen­yl)-*N*-(2-iodo­phen­yl)acetamide and 2-(4-chloro­phen­yl)-*N*-(pyrazin-2-yl)acetamide

**DOI:** 10.1107/S2056989016012512

**Published:** 2016-08-09

**Authors:** Badiadka Narayana, Hemmige S. Yathirajan, Ravindranath Rathore, Christopher Glidewell

**Affiliations:** a Department of Chemistry, Mangalore University, Mangalagangothri 574 199, India; b Department of Studies in Chemistry, University of Mysore, Manasagangotri, Mysuru 570 006, India; c Centre for Biological Sciences (Bioinformatics), Central University of South Bihar, BIT Campus, PO, B.V. College, Patna 800 014, Bihar State, India; d School of Chemistry, University of St Andrews, Fife KY16 9ST, Scotland

**Keywords:** crystal structure, C,N-disubstituted acetamides, mol­ecular structure, mol­ecular conformation, hydrogen bonding, C—halogen⋯π bonding

## Abstract

In the crystal of 2-(4-chloro­phen­yl)-*N*-(2-iodo­phen­yl)acetamide, C_14_H_11_ClINO, mol­ecules are linked by a combination of N—H⋯O and C—H⋯O hydrogen bonds to form a *C*(4)*C*(4)[*R*_2_^1^(7)] chain of rings and chains of this type are linked by a combination of C—Cl⋯π(arene) and C—I⋯π(arene) inter­actions to form deeply puckered twofold inter­woven sheets. In the crystal of 2-(4-chloro­phen­yl)-*N*-(pyrazin-2-yl)acetamide, C_12_H_10_ClN_3_O, mol­ecules are linked into complex sheets by N—H⋯N, C—H⋯N and C—H⋯O hydrogen bonds, and by C—H⋯π(arene) inter­actions.

## Abstract 2

A combination of N—H⋯O and C—H⋯O hydrogen bonds together with C—Cl⋯π(arene) and C—I⋯π(arene) inter­actions links the mol­ecules of 2-(4-chloro­phen­yl)-*N*-(2-iodo­phen­yl)acetamide into twofold inter­woven sheets, and the mol­ecules of 2-(4-chloro­phen­yl)-*N*-(pyrazin-2-yl)acetamide are linked into complex sheets built solely from hydrogen bonds.

## Chemical context   

Substituted acetamides of the type *R* ^1^CH_2_CONH*R* ^2^, where *R* ^1^ and *R* ^2^ are aromatic or hetero-aromatic substituents, are of inter­est as they have some resemblance to benzyl penicillins (Pitt, 1952 ▸; Csöregh & Palm, 1977 ▸; Kojić-Prodić & Rużoć-Toroš, 1978 ▸; Mijin & Marinković, 2006 ▸; Mijin *et al.*, 2008 ▸). Here we report on the mol­ecular structures and supra­molecular assembly of two such amides, compounds (I) and (II). The compounds were prepared by the reaction between (4-chloro­phen­yl)acetic acid and either 2-iodo­aniline for (I), or 2-amino­pyrazine for (II), using 1-ethyl-3-(3-di­methyl­amino­prop­yl)-carbodi­imide hydro­chloride as the coupling agent.

## Structural commentary   

**Figure 1:**
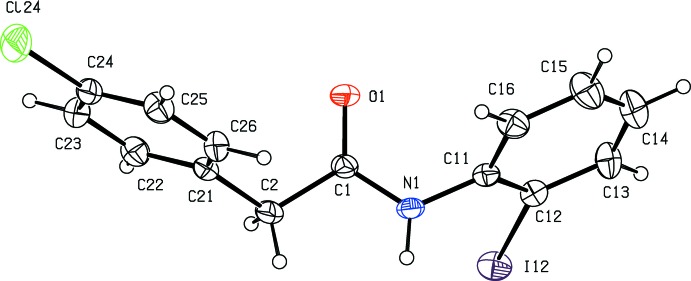
The mol­ecular structure of compound (I), showing the atom-labelling scheme. Displacement ellipsoids are drawn at the 30% probability level.

The mol­ecular conformations of compounds (I) and (II), illustrated in Figs. 1 ▸ and 2 ▸, respectively, can be defined in terms of the torsional angles N1—C1—C2—C21, 141.8 (3) and 129.22 (18)° respectively, and by the dihedral angles between the central spacer unit, atoms N1,C1,O1,C2, and the two independent rings. The dihedral angles to the chlorinated ring (C21–C26) are 80.02 (11) and 61.74 (6)° in (I) and (II); those to the iodinated ring in (I) and the pyrazinyl ring in (II) are 67.48 (11) and 5.86 (11)°, respectively. This difference is probably associated with the participation in the inter­molecular hydrogen bond of both N atoms of the pyrazinyl ring in (II), as discussed below. The mol­ecules of (I) and (II) do not therefore exhibit any inter­nal symmetry, so that they are conformationally chiral: the centrosymmetric space groups confirm that each compound has crystallized as a conformational racemate.
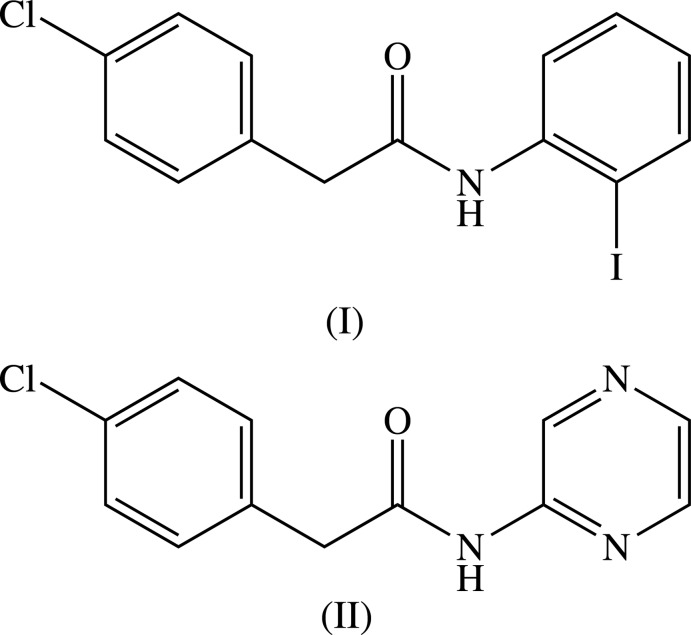


In the pyrazine ring of compound (II) the four independent C—N distances span a range of only *ca* 0.01 Å, indicating that this ring is fully aromatic.

## Supra­molecular inter­actions   

**Figure 3:**
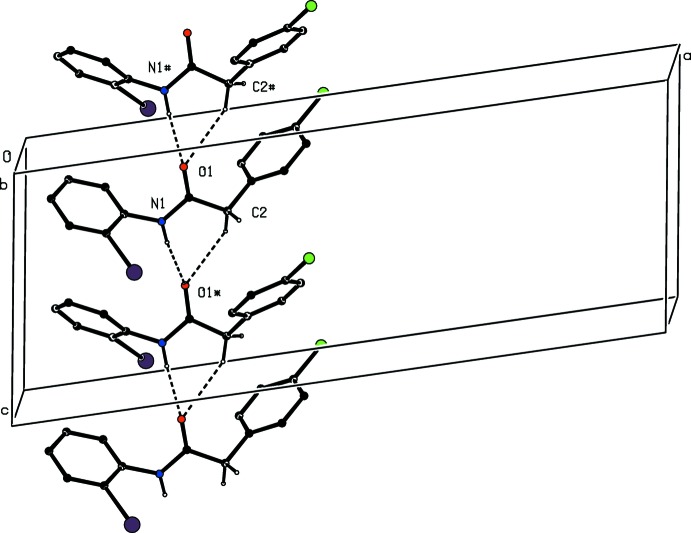
Part of the crystal structure of compound (I) showing the formation of a hydrogen-bonded chain of rings running parallel to the [001] direction. Hydrogen bonds are shown as dashed lines and, for the sake of clarity, the H atoms bonded to the C atoms which are not involved in the motif shown have been omitted. The atoms marked with an asterisk (*) or a hash (#) are at the symmetry positions (*x*, 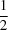 − *y*, 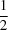 + *z*) and (*x*, 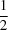 − *y*, −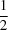 + *z*), respectively.

**Figure 4:**
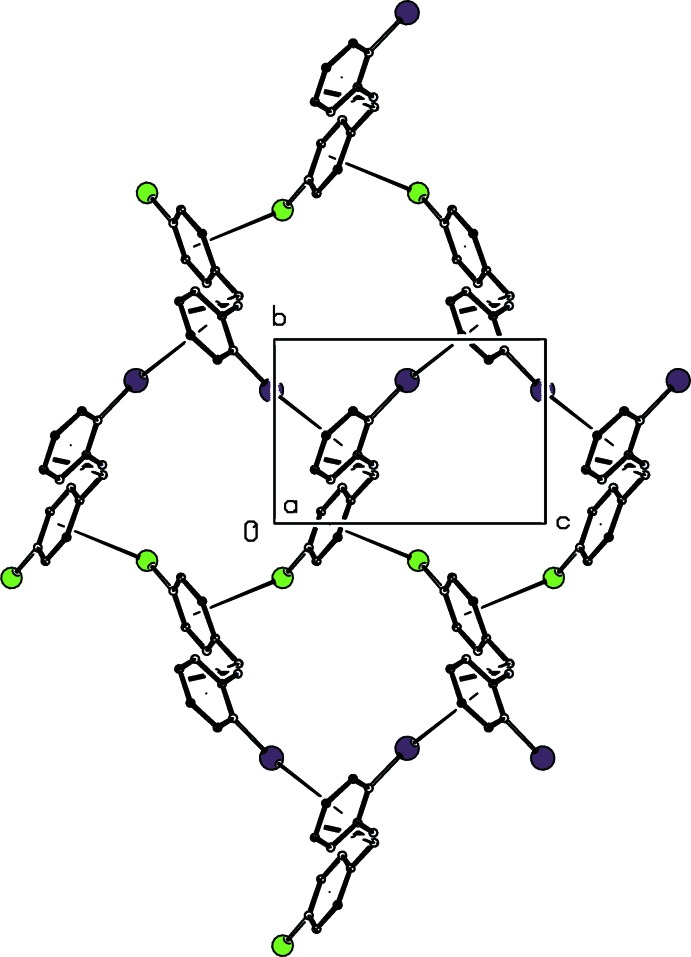
A projection down [100] of part of the crystal structure of compound (I) showing the formation of a sheet built from C—Cl⋯π(arene) and C—I⋯π(arene) inter­actions, shown as thin tapered lines. For the sake of clarity, the H atoms have all been omitted.

The hydrogen-bonded assembly in compound (I) is very simple: a combination of N—H⋯O and C—H⋯O hydrogen bonds (Table 1 ▸) links the mol­ecules into a *C*(4)*C*(4)[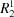
(6)] chain of rings. This chain contains mol­ecules which are related by a *c*-glide plane, producing a chain running parallel to the [001] direction (Fig. 3 ▸). There is also a C—H⋯π(arene) contact in compound (I) (Table 1 ▸), lying within the [001] chain, but the dimensions make it unlikely that this has any structural significance. Two chains of this type, which are related to one another by inversion, pass through each unit cell, and a combination of C—I⋯π(arene) and C—Cl⋯π(arene) inter­actions links the chains into a sheet in the form of a (4,4) net lying parallel to (100) (Fig. 4 ▸). The dimensions of these inter­actions are: for C12—I12⋯*Cg*1^i^ [symmetry code: (i) *x*, 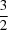 − *y*, 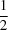 + *z*, where *Cg*1 represents the centroid of the C11–C16] ring, I⋯*Cg* 3.7977 (14), C⋯*Cg* 5.082 (3) Å and C—I⋯*Cg* 116.34 (8)°; for C24—Cl24⋯*Cg*2^ii^ [symmetry code: (ii) *x*, −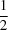 − *y*, −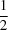 + *z*, where *Cg*2 represents the centroid of the C21–C26 ring], Cl⋯*Cg* 3.4557 (8), C⋯*Cg* 4.504 (3) Å and C—Cl⋯*Cg* 116.19 (11)°. The metrics of the C—Cl⋯*Cg* inter­action are well within the normal range, as deduced using database analysis (Imai *et al.*, 2008 ▸).

**Figure 5:**
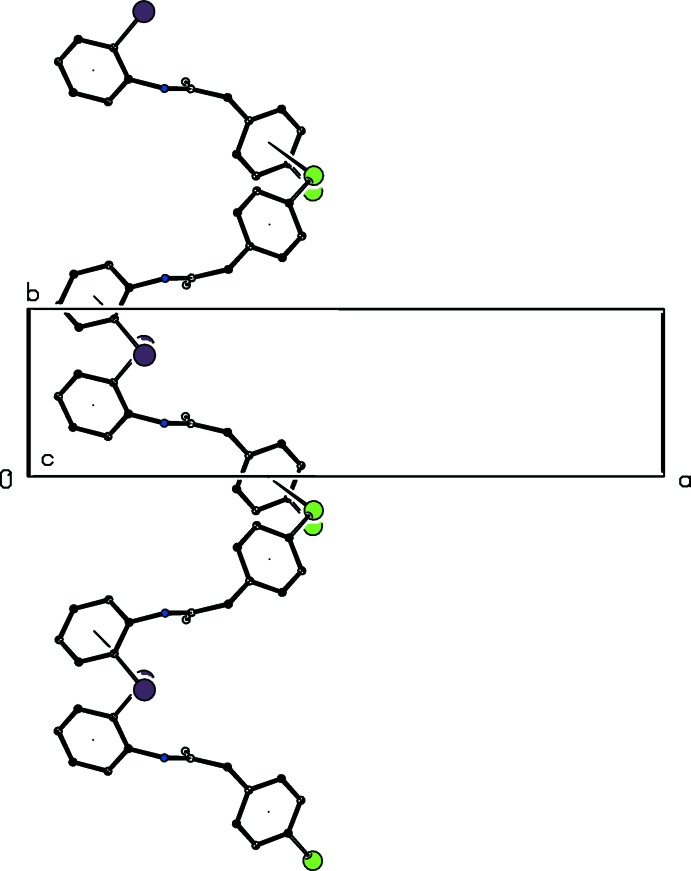
A projection down [001] of one of the (100) sheets in the crystal structure of compound (I) showing the deep puckering of the sheet enabling inter­weaving. The C—*X*⋯π(arene) inter­actions (*X* = Cl or I) are shown as thin tapered lines, and for the sake of clarity, the H atoms have all been omitted.

Because the repeat unit of this sheet in the [010] direction spans two unit cells, there are in fact two such sheets present, related to one another by a unit translation along [010]: the deep puckering of the sheets (Fig. 5 ▸) means that the two independent sheets are inter­woven. The structure of (I) also contains a short I⋯O contact with dimension I12⋯O1^i^ 3.058 (2) Å and C12—I12⋯O1^i^ 170.88 (8)° [symmetry code: (i) *x*, 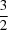 − *y*, 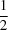 + *z*] which complements the C—Cl⋯*Cg* contact. The I⋯O distance here is significantly shorter than the sum of the van der Waals radii, 3.56 Å (Rowland & Taylor, 1996 ▸), or 3.30 Å if account is taken of the polar flattening model (Nyburg & Faerman, 1985 ▸).

**Figure 6:**
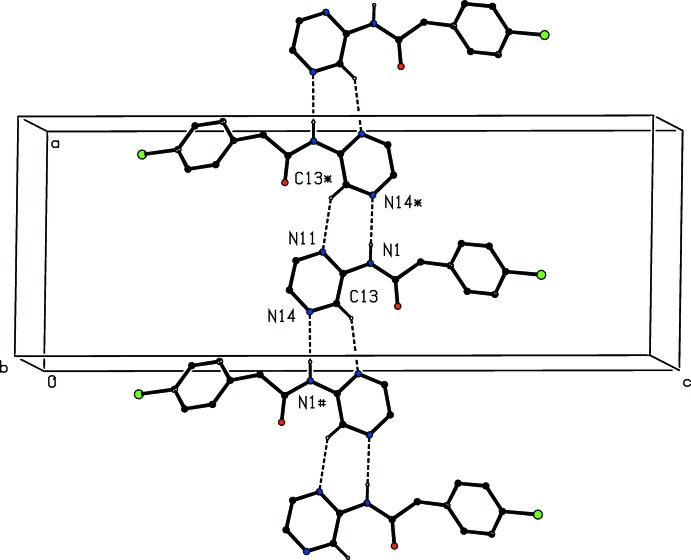
Part of the crystal structure of compound (II) showing the formation of a hydrogen-bonded chain of rings running parallel to the [010] direction and built from N—H⋯N and C—H⋯N hydrogen bonds, shown as dashed lines. For the sake of clarity, the C-bound H atoms which are not involved in the motifs shown have been omitted. The atoms marked with an asterisk (*) or a hash (#) are at the symmetry positions (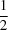 + *x*, 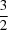 − *y*, 1 − *z*) and (−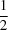 + *x*, 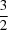 − *y*, 1 − *z*), respectively.

**Figure 7:**
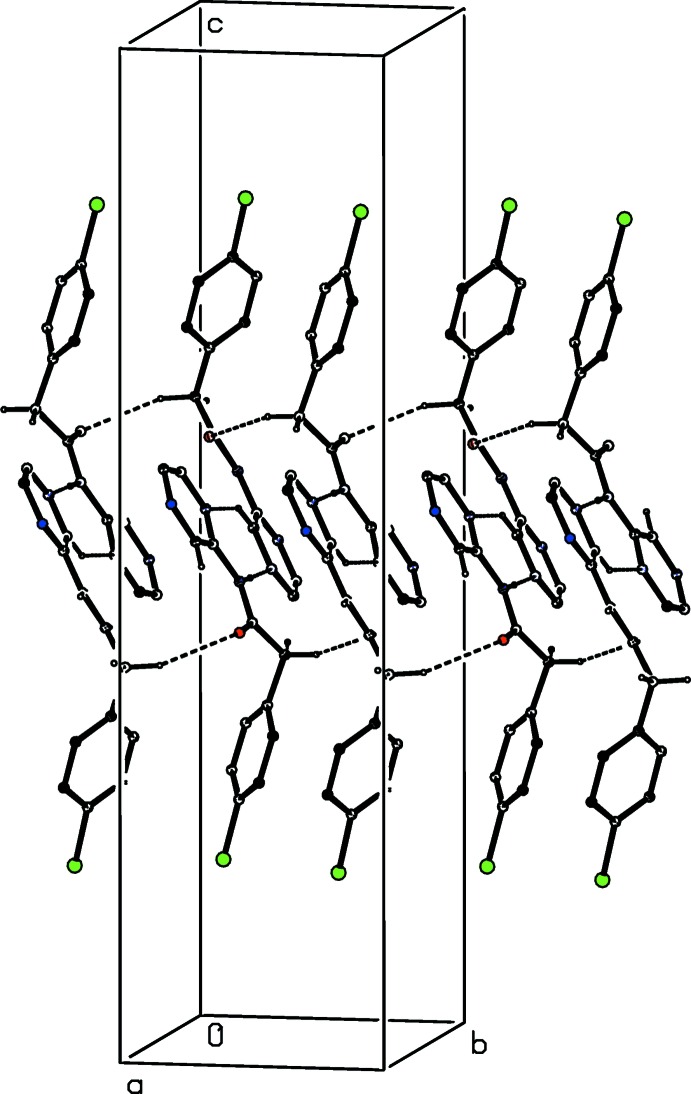
Part of the crystal structure of compound (II) showing the formation of a hydrogen-bonded ribbon of 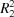
(7) and 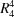
(22) rings running parallel to the [100] direction and built from N—H⋯N, C—H⋯N and C—H⋯O hydrogen bonds, shown as dashed lines. For the sake of clarity, the C-bound H atoms which are not involved in the motifs shown have been omitted.

The hydrogen-bonded supra­molecular assembly in compound (II) is more complex than that in compound (I): mol­ecules of (II) are linked into complex sheets by a combin­ation of N—H⋯N, C—H⋯N and C—H⋯O hydrogen bonds, weakly augmented by two C—H⋯π(arene) hydrogen bonds (Table 2 ▸): hydrogen bonds of N—H⋯O type, often observed in the structures of amides, are absent, however. The formation of this structure can readily be analysed in terms of two simple sub-structures in one- and two-dimensions (Ferguson *et al.*, 1998*a* ▸,*b* ▸; Gregson *et al.*, 2000 ▸). In the simpler of the sub-structures, a combination of N—H⋯N and C—H⋯N hydrogen bonds links mol­ecules which are related by the 2_1_ screw axis along (*x*, 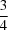, 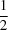) into a *C*(4)*C*(5)[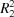
(7)] chain of rings running parallel to the [100] direction (Fig. 6 ▸). A more complex one-dimensional sub-structure results from the combination of the N—H⋯N, C—H⋯N and C—H⋯O hydrogen bonds, in the form of a ribbon containing alternating 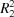
(7) and 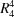
(22) rings (Fig. 7 ▸). The combination of these two chains along [100] and [010] generates a sheet lying parallel to (001) in the domain 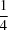 < *z* < 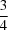, and a second such sheet, related to the first by inversion, lies in the domain 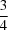 < *z* < 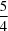. The C—H⋯π(arene) inter­actions both lie within the sheet.

## Database survey   

The structures of a number of 2-aryl-*N*-aryl acetamides related to compounds (I) and (II) have been reported recently. We note in particular the structure of 2-(4-chloro­phen­yl)-*N*-(2,6-di­methyl­phen­yl)acetamide (III) (Narayana *et al.*, 2016 ▸), where the mol­ecules are linked by a combination of N—H⋯O and C—H⋯O hydrogen bonds to form a *C*(4)*C*(4)[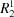
(7)] chain of rings very much like that in compound (I), except that the mol­ecules comprising the chain in (III) are related by translation along [100], whereas those in (I) are related by a *c*-glide plane. Other recently reported structures include those of *N*-(4-bromo­phen­yl)-2-(4-chloro­phen­yl)acetamide (IV) (Fun, Shahani *et al.*, 2012 ▸), 2-(4-bromo­phen­yl)-*N*-(pyrazin-2-yl)acetamide (V) (Nayak *et al.*, 2013 ▸) and 2-(4-chloro­phen­yl)-*N*-(2,6-di­methyl­phen­yl)acetamide (VI) (Fun, Quah *et al.*, 2012 ▸), which are related to compounds (I)–(III), respectively. In addition, the structures of some compounds related to (I), but carrying more than one substituent in the *N*-aryl ring have been reported (Praveen *et al.*, 2013*a* ▸,*b* ▸; Nayak *et al.*, 2014 ▸).

## Synthesis and crystallization   

For the synthesis of compounds (I) and (II), equimolar qu­anti­ties (1.0 mmol of each component) of (4-chloro­phen­yl)acetic acid and either 2-iodo­aniline for (I), or 2-amino­pyrazine for (II), were dissolved in di­chloro­methane (20 ml) in the presence of 1-ethyl-3-(3-di­methyl­amino­prop­yl)carbodi­imide hydro­chloride (0.01 mol) and tri­ethyl­amine (0.02 mol) at 273 K. The mixtures were stirred at 273 K for 3 h, and then poured with stirring into an excess of aqueous hydro­chloric acid (4 mol dm^−3^). The aqueous mixtures were exhaustively extracted with di­chloro­methane and in each case, the combined organic extracts were washed first with saturated aqueous sodium hydrogencarbonate solution and then with brine. The solutions were dried with anhydrous sodium sulfate and then the solvent was removed under reduced pressure, to give the products. Compound (I): yield 78%, m. p. 441–443 K; analysis found C 45.4, H 2.9, N 3.9%, C_14_H_11_ClINO requires C 45.2, H 3.0, N 3.8%. Compound (II): yield 85%, m. p. 421–423 K; analysis found C 58.3, H 4.2, N 16.9%, C_12_H_10_ClN_3_O requires C 58.2, H 4.1, N 17.0%. Crystals suitable for single-crystal X-ray diffraction analysis were grown by slow evaporation, at ambient temperature and in the presence of air, of solutions in di­chloro­methane.

## Refinement   

Crystal data, data collection and structure refinement details are summarized in Table 3 ▸. All H atoms were located in difference Fourier maps. The C-bound H atoms were then treated as riding atoms in geometrically idealized positions with C—H distances 0.93 Å (aromatic and hetero-aromatic) or 0.97 Å (CH_2_) and with *U*_iso_(H) = 1.2*U*_eq_(C). For the H atoms bonded to N atoms in compound (II), the atomic coordinates were refined with *U*_iso_(H) = 1.2*U*_eq_(N) giving the N—H distance shown in Table 2 ▸; an attempt to refine similarly the corresponding H-atom coordinates in compound (I) led to an unsatisfactorily low value, 0.74 (3) Å for the N—H distance, possibly associated with the presence of the strongly scattering iodene atom: accordingly this distance was thereafter fixed at 0.86 Å. A small number of low-angle reflections, which had been attenuated by the beam stop [(100) and (200) for (I); (002) for (II)] were omitted from the final cycles of refinement. In the final analysis of variance for compound (I), there was a large value, 4.245, of K = [mean(*F*_o_^2^)/mean(*F*_c_^2^)] for the group of 428 very weak reflections having *F*_c_/*F*_c_(max) in the range 0.000 < *F*_c_/*F*_c_(max) < 0.008.

## Supplementary Material

Crystal structure: contains datablock(s) global, I, II. DOI: 10.1107/S2056989016012512/su5316sup1.cif

Structure factors: contains datablock(s) I. DOI: 10.1107/S2056989016012512/su5316Isup2.hkl

Structure factors: contains datablock(s) II. DOI: 10.1107/S2056989016012512/su5316IIsup3.hkl

Supporting information file. DOI: 10.1107/S2056989016012512/su5316Isup4.cml

Supporting information file. DOI: 10.1107/S2056989016012512/su5316IIsup5.cml

CCDC references: 1497360, 1497359

Additional supporting information:  crystallographic information; 3D view; checkCIF report

## Figures and Tables

**Figure 2:**
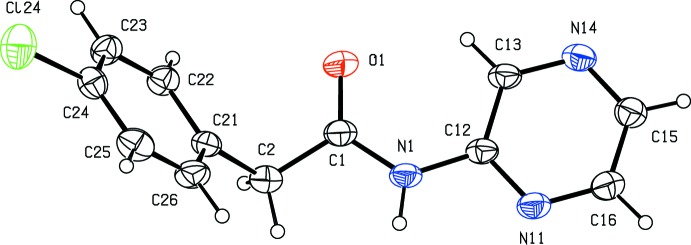
The mol­ecular structure of compound (II), showing the atom-labelling scheme. Displacement ellipsoids are drawn at the 30% probability level.

**Table 1:** Hydrogen-bond geometry (Å, °) for (I) *Cg*2 is the centroid of the C21–C26 ring.

*D*—H⋯*A*	*D*—H	H⋯*A*	*D*⋯*A*	*D*—H⋯*A*
N1—H1⋯O1^i^	0.86	2.06	2.908 (3)	167
C2—H2*A*⋯O1^i^	0.97	2.58	3.420 (4)	145
C2—H2*B*⋯*Cg*2^i^	0.97	2.99	3.589 (3)	121

Symmetry code: (i) 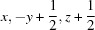.

**Table 2:** Hydrogen-bond geometry (Å, °) for (II) *Cg*2 is the centroid of the C21–C26 ring.

*D*—H⋯*A*	*D*—H	H⋯*A*	*D*⋯*A*	*D*—H⋯*A*
N1—H1⋯N14^i^	0.85 (2)	2.23 (2)	3.077 (2)	175 (2)
C2—H2*A*⋯O1^ii^	0.97	2.57	3.461 (3)	153
C13—H13⋯N11^iii^	0.93	2.50	3.277 (2)	142
C22—H22⋯*Cg*2^ii^	0.93	2.99	3.6416 (17)	129
C25—H25⋯*Cg*2^iv^	0.93	2.89	3.743 (2)	154

Symmetry codes: (i) 
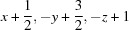
; (ii) 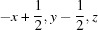; (iii) 
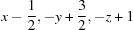
; (iv) 
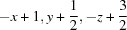
.

**Table 3:** Experimental details

	(I)	(II)
Crystal data		
Chemical formula	C_14_H_11_ClINO	C_12_H_10_ClN_3_O
*M*_r_	371.59	247.68
Crystal system, space group	Monoclinic, *P*2_1_/*c*	Orthorhombic, *P* *b* *c* *a*
Temperature (K)	295	295
*a*, *b*, *c* (Å)	24.001 (1), 6.2369 (3), 9.3266 (4)	10.7041 (4), 7.5724 (3), 28.6619 (11)
α, β, γ (°)	90, 99.621 (2), 90	90, 90, 90
*V* (Å^3^)	1376.48 (11)	2323.21 (15)
*Z*	4	8
Radiation type	Mo *K*α	Mo *K*α
μ (mm^−1^)	2.51	0.32
Crystal size (mm)	0.30 × 0.18 × 0.12	0.40 × 0.30 × 0.20

Data collection		
Diffractometer	Bruker APEXII area detector	Bruker APEXII area detector
Absorption correction	Multi-scan (*SADABS*; Sheldrick, 2003 ▸)	Multi-scan (*SADABS*; Sheldrick, 2003 ▸)
*T*_min_, *T*_max_	0.528, 0.740	0.739, 0.939
No. of measured, independent and observed [*I* > 2σ(*I*)] reflections	15082, 3960, 3183	24592, 3380, 2287
*R*_int_	0.026	0.029
(sin θ/λ)_max_ (Å^−1^)	0.703	0.703

Refinement		
*R*[*F* ^2^ > 2σ(*F* ^2^)], *wR*(*F* ^2^), *S*	0.037, 0.074, 1.07	0.047, 0.136, 1.02
No. of reflections	3960	3380
No. of parameters	163	157
H-atom treatment	H-atom parameters constrained	H-atom parameters constrained
Δρ_max_, Δρ_min_ (e Å^−3^)	1.49, −0.60	0.47, −0.51

Computer programs: *APEX2* and *SAINT-Plus* (Bruker, 2012 ▸), *SHELXS97* (Sheldrick, 2008 ▸), *SHELXL2014* (Sheldrick, 2015 ▸) and *PLATON* (Spek, 2009 ▸).

## supplementary crystallographic information

### (I) 2-(4-Chlorophenyl)-N-(2-iodophenyl)acetamide Crystal data

**Table:**

C_14_H_11_ClINO	*F*(000) = 720
*M**_r_* = 371.59	*D*_x_ = 1.793 Mg m^−^^3^
Monoclinic, *P*2_1_/*c*	Mo *K*α radiation, λ = 0.71073 Å
*a* = 24.001 (1) Å	Cell parameters from 5030 reflections
*b* = 6.2369 (3) Å	θ = 0.9–33.5°
*c* = 9.3266 (4) Å	µ = 2.51 mm^−^^1^
β = 99.621 (2)°	*T* = 295 K
*V* = 1376.48 (11) Å^3^	Block, colourless
*Z* = 4	0.30 × 0.18 × 0.12 mm

### (I) 2-(4-Chlorophenyl)-N-(2-iodophenyl)acetamide Data collection

**Table:**

Bruker APEXII area detector diffractometer	3960 independent reflections
Radiation source: fine-focus sealed tube	3183 reflections with *I* > 2σ(*I*)
Graphite monochromator	*R*_int_ = 0.026
φ and ω scans	θ_max_ = 30.0°, θ_min_ = 2.6°
Absorption correction: multi-scan (SADABS; Sheldrick, 2003)	*h* = −33→33
*T*_min_ = 0.528, *T*_max_ = 0.740	*k* = −8→8
15082 measured reflections	*l* = −13→9

### (I) 2-(4-Chlorophenyl)-N-(2-iodophenyl)acetamide Refinement

**Table:**

Refinement on *F*^2^	0 restraints
Least-squares matrix: full	Hydrogen site location: inferred from neighbouring sites
*R*[*F*^2^ > 2σ(*F*^2^)] = 0.037	H-atom parameters constrained
*wR*(*F*^2^) = 0.074	*w* = 1/[σ^2^(*F*_o_^2^) + (0.0189*P*)^2^ + 1.9949*P*] where *P* = (*F*_o_^2^ + 2*F*_c_^2^)/3
*S* = 1.07	(Δ/σ)_max_ = 0.001
3960 reflections	Δρ_max_ = 1.49 e Å^−^^3^
163 parameters	Δρ_min_ = −0.60 e Å^−^^3^

### (I) 2-(4-Chlorophenyl)-N-(2-iodophenyl)acetamide Special details

**Table:**

Geometry. All esds (except the esd in the dihedral angle between two l.s. planes) are estimated using the full covariance matrix. The cell esds are taken into account individually in the estimation of esds in distances, angles and torsion angles; correlations between esds in cell parameters are only used when they are defined by crystal symmetry. An approximate (isotropic) treatment of cell esds is used for estimating esds involving l.s. planes.

### (I) 2-(4-Chlorophenyl)-N-(2-iodophenyl)acetamide Fractional atomic coordinates and isotropic or equivalent isotropic displacement parameters (Å^2^)

**Table:**

	*x*	*y*	*z*	*U*_iso_*/*U*_eq_	
C1	0.25595 (11)	0.3149 (5)	0.2858 (3)	0.0344 (6)	
O1	0.24811 (9)	0.3574 (4)	0.1558 (2)	0.0496 (6)	
N1	0.21438 (10)	0.3174 (4)	0.3652 (2)	0.0340 (5)	
H1	0.2223	0.2839	0.4557	0.041*	
C2	0.31392 (12)	0.2634 (6)	0.3678 (3)	0.0445 (7)	
H2A	0.3101	0.1900	0.4574	0.053*	
H2B	0.3342	0.3962	0.3935	0.053*	
C11	0.15786 (11)	0.3726 (4)	0.3066 (3)	0.0325 (6)	
C12	0.13408 (11)	0.5596 (4)	0.3479 (3)	0.0350 (6)	
I12	0.18141 (2)	0.77640 (3)	0.48960 (2)	0.04554 (8)	
C13	0.07856 (13)	0.6103 (6)	0.2902 (4)	0.0506 (8)	
H13	0.0623	0.7349	0.3192	0.061*	
C14	0.04773 (14)	0.4773 (7)	0.1907 (4)	0.0610 (10)	
H14	0.0107	0.5131	0.1511	0.073*	
C15	0.07091 (15)	0.2923 (6)	0.1492 (5)	0.0626 (10)	
H15	0.0497	0.2025	0.0815	0.075*	
C16	0.12596 (14)	0.2378 (5)	0.2076 (4)	0.0456 (7)	
H16	0.1415	0.1106	0.1802	0.055*	
C21	0.34782 (11)	0.1252 (5)	0.2816 (3)	0.0363 (6)	
C22	0.39910 (13)	0.1937 (5)	0.2504 (4)	0.0441 (7)	
H22	0.4129	0.3279	0.2821	0.053*	
C23	0.43017 (13)	0.0644 (6)	0.1724 (4)	0.0472 (8)	
H23	0.4646	0.1119	0.1512	0.057*	
C24	0.41012 (12)	−0.1323 (6)	0.1268 (3)	0.0440 (7)	
Cl24	0.44931 (4)	−0.2968 (2)	0.03026 (13)	0.0754 (3)	
C25	0.35920 (13)	−0.2053 (5)	0.1558 (4)	0.0460 (7)	
H25	0.3457	−0.3397	0.1237	0.055*	
C26	0.32848 (12)	−0.0756 (6)	0.2333 (3)	0.0440 (7)	
H26	0.2940	−0.1241	0.2536	0.053*	

### (I) 2-(4-Chlorophenyl)-N-(2-iodophenyl)acetamide Atomic displacement parameters (Å^2^)

**Table:**

	*U*^11^	*U*^22^	*U*^33^	*U*^12^	*U*^13^	*U*^23^
C1	0.0379 (14)	0.0370 (15)	0.0273 (14)	0.0082 (11)	0.0026 (11)	−0.0040 (11)
O1	0.0484 (12)	0.0757 (16)	0.0244 (11)	0.0167 (11)	0.0056 (9)	0.0077 (11)
N1	0.0421 (12)	0.0387 (13)	0.0214 (11)	0.0094 (10)	0.0057 (9)	0.0008 (9)
C2	0.0408 (15)	0.065 (2)	0.0257 (14)	0.0123 (14)	−0.0011 (11)	−0.0074 (14)
C11	0.0379 (14)	0.0331 (14)	0.0268 (13)	0.0007 (11)	0.0059 (10)	0.0026 (11)
C12	0.0385 (14)	0.0322 (14)	0.0349 (15)	−0.0005 (11)	0.0082 (11)	−0.0002 (11)
I12	0.05868 (14)	0.03484 (11)	0.04157 (12)	−0.00133 (9)	0.00388 (9)	−0.00538 (9)
C13	0.0365 (16)	0.0478 (19)	0.067 (2)	0.0083 (13)	0.0081 (15)	−0.0018 (17)
C14	0.0366 (17)	0.068 (2)	0.075 (3)	−0.0006 (16)	−0.0011 (16)	0.002 (2)
C15	0.0490 (19)	0.064 (2)	0.068 (3)	−0.0141 (17)	−0.0083 (17)	−0.011 (2)
C16	0.0515 (18)	0.0405 (17)	0.0431 (18)	0.0003 (13)	0.0031 (14)	−0.0052 (14)
C21	0.0332 (13)	0.0469 (17)	0.0271 (14)	0.0082 (12)	0.0003 (10)	0.0029 (12)
C22	0.0397 (15)	0.0464 (18)	0.0441 (18)	−0.0023 (13)	0.0013 (13)	−0.0022 (14)
C23	0.0350 (15)	0.062 (2)	0.0460 (19)	−0.0005 (14)	0.0100 (13)	0.0001 (16)
C24	0.0380 (15)	0.060 (2)	0.0328 (16)	0.0157 (14)	0.0030 (12)	−0.0055 (14)
Cl24	0.0581 (5)	0.0985 (8)	0.0703 (7)	0.0263 (5)	0.0122 (5)	−0.0270 (6)
C25	0.0452 (17)	0.0417 (17)	0.0487 (19)	0.0025 (13)	0.0008 (14)	−0.0069 (14)
C26	0.0304 (14)	0.0551 (19)	0.0466 (18)	−0.0005 (13)	0.0063 (12)	0.0012 (15)

### (I) 2-(4-Chlorophenyl)-N-(2-iodophenyl)acetamide Geometric parameters (Å, º)

**Table:**

C1—O1	1.225 (3)	C14—H14	0.9300
C1—N1	1.338 (4)	C15—C16	1.385 (5)
C1—C2	1.506 (4)	C15—H15	0.9300
N1—C11	1.418 (3)	C16—H16	0.9300
N1—H1	0.8600	C21—C22	1.379 (4)
C2—C21	1.507 (4)	C21—C26	1.385 (4)
C2—H2A	0.9700	C22—C23	1.385 (5)
C2—H2B	0.9700	C22—H22	0.9300
C11—C12	1.381 (4)	C23—C24	1.360 (5)
C11—C16	1.381 (4)	C23—H23	0.9300
C12—C13	1.388 (4)	C24—C25	1.373 (5)
C12—I12	2.089 (3)	C24—Cl24	1.741 (3)
C13—C14	1.366 (5)	C25—C26	1.378 (4)
C13—H13	0.9300	C25—H25	0.9300
C14—C15	1.365 (5)	C26—H26	0.9300
			
O1—C1—N1	122.7 (3)	C14—C15—C16	120.2 (3)
O1—C1—C2	121.7 (3)	C14—C15—H15	119.9
N1—C1—C2	115.6 (2)	C16—C15—H15	119.9
C1—N1—C11	122.9 (2)	C11—C16—C15	120.0 (3)
C1—N1—H1	118.5	C11—C16—H16	120.0
C11—N1—H1	118.5	C15—C16—H16	120.0
C1—C2—C21	112.7 (2)	C22—C21—C26	118.3 (3)
C1—C2—H2A	109.0	C22—C21—C2	121.0 (3)
C21—C2—H2A	109.0	C26—C21—C2	120.6 (3)
C1—C2—H2B	109.0	C21—C22—C23	120.6 (3)
C21—C2—H2B	109.0	C21—C22—H22	119.7
H2A—C2—H2B	107.8	C23—C22—H22	119.7
C12—C11—C16	119.4 (3)	C24—C23—C22	119.8 (3)
C12—C11—N1	120.7 (2)	C24—C23—H23	120.1
C16—C11—N1	119.8 (3)	C22—C23—H23	120.1
C11—C12—C13	119.9 (3)	C23—C24—C25	121.2 (3)
C11—C12—I12	121.1 (2)	C23—C24—Cl24	120.0 (3)
C13—C12—I12	118.9 (2)	C25—C24—Cl24	118.8 (3)
C14—C13—C12	120.1 (3)	C24—C25—C26	118.7 (3)
C14—C13—H13	120.0	C24—C25—H25	120.6
C12—C13—H13	120.0	C26—C25—H25	120.6
C15—C14—C13	120.4 (3)	C25—C26—C21	121.5 (3)
C15—C14—H14	119.8	C25—C26—H26	119.3
C13—C14—H14	119.8	C21—C26—H26	119.3
			
O1—C1—N1—C11	−1.1 (5)	N1—C11—C16—C15	−179.4 (3)
C2—C1—N1—C11	177.0 (3)	C14—C15—C16—C11	−1.0 (6)
O1—C1—C2—C21	−40.1 (4)	C1—C2—C21—C22	121.2 (3)
N1—C1—C2—C21	141.8 (3)	C1—C2—C21—C26	−59.7 (4)
C1—N1—C11—C12	−111.7 (3)	C26—C21—C22—C23	0.2 (5)
C1—N1—C11—C16	68.6 (4)	C2—C21—C22—C23	179.3 (3)
C16—C11—C12—C13	0.1 (4)	C21—C22—C23—C24	−0.4 (5)
N1—C11—C12—C13	−179.6 (3)	C22—C23—C24—C25	0.4 (5)
C16—C11—C12—I12	−177.1 (2)	C22—C23—C24—Cl24	−179.3 (3)
N1—C11—C12—I12	3.2 (4)	C23—C24—C25—C26	−0.3 (5)
C11—C12—C13—C14	−1.1 (5)	Cl24—C24—C25—C26	179.4 (3)
I12—C12—C13—C14	176.2 (3)	C24—C25—C26—C21	0.2 (5)
C12—C13—C14—C15	1.1 (6)	C22—C21—C26—C25	−0.1 (5)
C13—C14—C15—C16	0.0 (6)	C2—C21—C26—C25	−179.2 (3)
C12—C11—C16—C15	0.9 (5)		

### (I) 2-(4-Chlorophenyl)-N-(2-iodophenyl)acetamide Hydrogen-bond geometry (Å, º)

Cg2 is the centroid of the C21–C26 ring.

**Table:**

*D*—H···*A*	*D*—H	H···*A*	*D*···*A*	*D*—H···*A*
N1—H1···O1^i^	0.86	2.06	2.908 (3)	167
C2—H2*A*···O1^i^	0.97	2.58	3.420 (4)	145
C2—H2*B*···*Cg*2^i^	0.97	2.99	3.589 (3)	121

Symmetry code: (i) *x*, −*y*+1/2, *z*+1/2.

### (II) 2-(4-Chlorophenyl)-N-(pyrazin-2-yl)acetamide Crystal data

**Table:**

C_12_H_10_ClN_3_O	*D*_x_ = 1.416 Mg m^−^^3^
*M**_r_* = 247.68	Mo *K*α radiation, λ = 0.71073 Å
Orthorhombic, *P**b**c**a*	Cell parameters from 3760 reflections
*a* = 10.7041 (4) Å	θ = 1.4–32.3°
*b* = 7.5724 (3) Å	µ = 0.32 mm^−^^1^
*c* = 28.6619 (11) Å	*T* = 295 K
*V* = 2323.21 (15) Å^3^	Block, colourless
*Z* = 8	0.40 × 0.30 × 0.20 mm
*F*(000) = 1024	

### (II) 2-(4-Chlorophenyl)-N-(pyrazin-2-yl)acetamide Data collection

**Table:**

Bruker APEXII area detector diffractometer	3380 independent reflections
Radiation source: fine-focus sealed tube	2287 reflections with *I* > 2σ(*I*)
Graphite monochromator	*R*_int_ = 0.029
φ and ω scans	θ_max_ = 30.0°, θ_min_ = 2.4°
Absorption correction: multi-scan (SADABS; Sheldrick, 2003)	*h* = −15→14
*T*_min_ = 0.739, *T*_max_ = 0.939	*k* = −9→10
24592 measured reflections	*l* = −40→40

### (II) 2-(4-Chlorophenyl)-N-(pyrazin-2-yl)acetamide Refinement

**Table:**

Refinement on *F*^2^	0 restraints
Least-squares matrix: full	Hydrogen site location: mixed
*R*[*F*^2^ > 2σ(*F*^2^)] = 0.047	H-atom parameters constrained
*wR*(*F*^2^) = 0.136	*w* = 1/[σ^2^(*F*_o_^2^) + (0.0547*P*)^2^ + 0.9776*P*] where *P* = (*F*_o_^2^ + 2*F*_c_^2^)/3
*S* = 1.02	(Δ/σ)_max_ < 0.001
3380 reflections	Δρ_max_ = 0.47 e Å^−^^3^
157 parameters	Δρ_min_ = −0.51 e Å^−^^3^

### (II) 2-(4-Chlorophenyl)-N-(pyrazin-2-yl)acetamide Special details

**Table:**

Geometry. All esds (except the esd in the dihedral angle between two l.s. planes) are estimated using the full covariance matrix. The cell esds are taken into account individually in the estimation of esds in distances, angles and torsion angles; correlations between esds in cell parameters are only used when they are defined by crystal symmetry. An approximate (isotropic) treatment of cell esds is used for estimating esds involving l.s. planes.

### (II) 2-(4-Chlorophenyl)-N-(pyrazin-2-yl)acetamide Fractional atomic coordinates and isotropic or equivalent isotropic displacement parameters (Å^2^)

**Table:**

	*x*	*y*	*z*	*U*_iso_*/*U*_eq_	
C1	0.34192 (16)	0.6027 (3)	0.57916 (6)	0.0514 (4)	
O1	0.23005 (12)	0.6165 (3)	0.58396 (5)	0.0760 (5)	
N1	0.40586 (13)	0.6699 (2)	0.54222 (5)	0.0488 (4)	
H1	0.484 (2)	0.654 (3)	0.5421 (7)	0.059*	
C2	0.42190 (17)	0.5037 (3)	0.61387 (7)	0.0561 (5)	
H2A	0.4031	0.3787	0.6118	0.067*	
H2B	0.5091	0.5195	0.6057	0.067*	
N11	0.44426 (13)	0.8104 (2)	0.47344 (6)	0.0522 (4)	
C12	0.35844 (15)	0.7566 (2)	0.50359 (6)	0.0430 (4)	
C13	0.23119 (15)	0.7832 (3)	0.49531 (6)	0.0472 (4)	
H13	0.1730	0.7436	0.5170	0.057*	
N14	0.19234 (13)	0.8640 (2)	0.45707 (5)	0.0507 (4)	
C15	0.27929 (17)	0.9194 (3)	0.42717 (7)	0.0531 (4)	
H15	0.2550	0.9773	0.4000	0.064*	
C16	0.40366 (18)	0.8925 (3)	0.43564 (7)	0.0562 (5)	
H16	0.4617	0.9335	0.4141	0.067*	
C21	0.40218 (15)	0.5639 (2)	0.66329 (6)	0.0440 (4)	
C22	0.29842 (16)	0.5106 (2)	0.68824 (6)	0.0470 (4)	
H22	0.2383	0.4408	0.6738	0.056*	
C23	0.28265 (16)	0.5594 (2)	0.73413 (6)	0.0492 (4)	
H23	0.2124	0.5228	0.7506	0.059*	
C24	0.37099 (18)	0.6618 (2)	0.75533 (6)	0.0486 (4)	
Cl24	0.35395 (7)	0.71602 (9)	0.81390 (2)	0.0808 (2)	
C25	0.47361 (18)	0.7207 (3)	0.73145 (8)	0.0575 (5)	
H25	0.5325	0.7925	0.7460	0.069*	
C26	0.48787 (16)	0.6715 (3)	0.68544 (7)	0.0555 (5)	
H26	0.5570	0.7118	0.6689	0.067*	

### (II) 2-(4-Chlorophenyl)-N-(pyrazin-2-yl)acetamide Atomic displacement parameters (Å^2^)

**Table:**

	*U*^11^	*U*^22^	*U*^33^	*U*^12^	*U*^13^	*U*^23^
C1	0.0365 (8)	0.0669 (12)	0.0507 (9)	0.0063 (8)	0.0034 (7)	−0.0025 (8)
O1	0.0359 (7)	0.1235 (14)	0.0685 (9)	0.0117 (8)	0.0105 (6)	0.0210 (9)
N1	0.0278 (6)	0.0693 (10)	0.0492 (8)	0.0028 (7)	0.0001 (6)	−0.0025 (7)
C2	0.0434 (9)	0.0685 (13)	0.0563 (10)	0.0119 (9)	0.0050 (8)	0.0023 (9)
N11	0.0331 (7)	0.0684 (11)	0.0549 (8)	0.0003 (7)	0.0030 (6)	−0.0011 (8)
C12	0.0315 (7)	0.0526 (9)	0.0449 (8)	−0.0001 (7)	−0.0004 (6)	−0.0100 (7)
C13	0.0303 (7)	0.0622 (11)	0.0489 (9)	0.0015 (7)	−0.0005 (6)	−0.0074 (8)
N14	0.0373 (7)	0.0616 (10)	0.0532 (8)	0.0057 (7)	−0.0032 (6)	−0.0080 (7)
C15	0.0474 (10)	0.0615 (12)	0.0503 (9)	0.0049 (9)	−0.0019 (7)	−0.0021 (9)
C16	0.0437 (9)	0.0686 (13)	0.0563 (10)	0.0005 (9)	0.0070 (8)	0.0014 (9)
C21	0.0337 (7)	0.0449 (9)	0.0534 (9)	0.0055 (7)	0.0011 (7)	0.0054 (7)
C22	0.0401 (8)	0.0454 (10)	0.0556 (10)	−0.0074 (7)	−0.0003 (7)	0.0003 (8)
C23	0.0453 (9)	0.0463 (10)	0.0560 (9)	−0.0047 (8)	0.0067 (7)	0.0046 (8)
C24	0.0536 (10)	0.0421 (9)	0.0499 (9)	0.0035 (8)	−0.0046 (8)	0.0009 (7)
Cl24	0.1048 (5)	0.0848 (5)	0.0529 (3)	−0.0046 (4)	−0.0051 (3)	−0.0082 (3)
C25	0.0456 (10)	0.0533 (11)	0.0738 (12)	−0.0094 (8)	−0.0114 (9)	−0.0054 (9)
C26	0.0341 (8)	0.0615 (12)	0.0709 (12)	−0.0066 (8)	0.0044 (8)	0.0059 (10)

### (II) 2-(4-Chlorophenyl)-N-(pyrazin-2-yl)acetamide Geometric parameters (Å, º)

**Table:**

C1—O1	1.210 (2)	C15—C16	1.368 (3)
C1—N1	1.360 (2)	C15—H15	0.9300
C1—C2	1.511 (3)	C16—H16	0.9300
N1—C12	1.384 (2)	C21—C22	1.381 (2)
N1—H1	0.85 (2)	C21—C26	1.382 (3)
C2—C21	1.503 (3)	C22—C23	1.377 (3)
C2—H2A	0.9700	C22—H22	0.9300
C2—H2B	0.9700	C23—C24	1.366 (3)
N11—C16	1.323 (3)	C23—H23	0.9300
N11—C12	1.325 (2)	C24—C25	1.369 (3)
C12—C13	1.397 (2)	C24—Cl24	1.7378 (19)
C13—N14	1.322 (2)	C25—C26	1.379 (3)
C13—H13	0.9300	C25—H25	0.9300
N14—C15	1.333 (2)	C26—H26	0.9300
			
O1—C1—N1	123.67 (18)	C16—C15—H15	119.4
O1—C1—C2	121.92 (18)	N11—C16—C15	122.33 (18)
N1—C1—C2	114.39 (15)	N11—C16—H16	118.8
C1—N1—C12	128.03 (14)	C15—C16—H16	118.8
C1—N1—H1	116.6 (14)	C22—C21—C26	117.92 (17)
C12—N1—H1	115.3 (14)	C22—C21—C2	120.83 (17)
C21—C2—C1	112.98 (15)	C26—C21—C2	121.24 (16)
C21—C2—H2A	109.0	C23—C22—C21	121.00 (17)
C1—C2—H2A	109.0	C23—C22—H22	119.5
C21—C2—H2B	109.0	C21—C22—H22	119.5
C1—C2—H2B	109.0	C24—C23—C22	119.51 (17)
H2A—C2—H2B	107.8	C24—C23—H23	120.2
C16—N11—C12	116.79 (15)	C22—C23—H23	120.2
N11—C12—N1	114.40 (14)	C23—C24—C25	121.21 (18)
N11—C12—C13	121.38 (16)	C23—C24—Cl24	119.43 (15)
N1—C12—C13	124.19 (16)	C25—C24—Cl24	119.35 (15)
N14—C13—C12	120.94 (16)	C24—C25—C26	118.61 (18)
N14—C13—H13	119.5	C24—C25—H25	120.7
C12—C13—H13	119.5	C26—C25—H25	120.7
C13—N14—C15	117.31 (15)	C25—C26—C21	121.70 (17)
N14—C15—C16	121.23 (18)	C25—C26—H26	119.2
N14—C15—H15	119.4	C21—C26—H26	119.2
			
O1—C1—N1—C12	−2.8 (3)	N14—C15—C16—N11	0.3 (3)
C2—C1—N1—C12	175.70 (18)	C1—C2—C21—C22	77.6 (2)
O1—C1—C2—C21	−52.2 (3)	C1—C2—C21—C26	−103.5 (2)
N1—C1—C2—C21	129.22 (18)	C26—C21—C22—C23	−1.7 (3)
C16—N11—C12—N1	179.33 (17)	C2—C21—C22—C23	177.25 (17)
C16—N11—C12—C13	1.0 (3)	C21—C22—C23—C24	−0.1 (3)
C1—N1—C12—N11	178.81 (18)	C22—C23—C24—C25	1.7 (3)
C1—N1—C12—C13	−2.9 (3)	C22—C23—C24—Cl24	−177.50 (14)
N11—C12—C13—N14	−0.3 (3)	C23—C24—C25—C26	−1.4 (3)
N1—C12—C13—N14	−178.47 (17)	Cl24—C24—C25—C26	177.80 (16)
C12—C13—N14—C15	−0.4 (3)	C24—C25—C26—C21	−0.5 (3)
C13—N14—C15—C16	0.4 (3)	C22—C21—C26—C25	2.0 (3)
C12—N11—C16—C15	−1.0 (3)	C2—C21—C26—C25	−176.93 (19)

### (II) 2-(4-Chlorophenyl)-N-(pyrazin-2-yl)acetamide Hydrogen-bond geometry (Å, º)

Cg2 is the centroid of the C21–C26 ring.

**Table:**

*D*—H···*A*	*D*—H	H···*A*	*D*···*A*	*D*—H···*A*
N1—H1···N14^i^	0.85 (2)	2.23 (2)	3.077 (2)	175 (2)
C2—H2*A*···O1^ii^	0.97	2.57	3.461 (3)	153
C13—H13···N11^iii^	0.93	2.50	3.277 (2)	142
C22—H22···*Cg*2^ii^	0.93	2.99	3.6416 (17)	129
C25—H25···*Cg*2^iv^	0.93	2.89	3.743 (2)	154

Symmetry codes: (i) *x*+1/2, −*y*+3/2, −*z*+1; (ii) −*x*+1/2, *y*−1/2, *z*; (iii) *x*−1/2, −*y*+3/2, −*z*+1; (iv) −*x*+1, *y*+1/2, −*z*+3/2.
